# Orthopedic resident education on postoperative pain control: bridging knowledge gaps to enhance patient safety

**DOI:** 10.5116/ijme.5a91.2f7f

**Published:** 2018-03-09

**Authors:** Lindsay L. Warner, Paul A. Warner, Jason S. Eldrige

**Affiliations:** 1 Department of Anesthesiology and Perioperative Medicine, Mayo Clinic, Rochester, USA

**Keywords:** Orthopedic resident education, postoperative pain control, knowledge gaps, patient safety

## Introduction

As the United States population continues to age, the number of orthopedic procedures continues to grow.^1^ Not infrequently, many of these patients who are admitted for orthopedic surgical procedures are already on complex pain regimens preoperatively.  This is due not only to the increasing comorbid state of our aging population but also the wide variety of analgesic management strategies employed throughout the nation amid chronic opioid use.^2^ Expanding surgical volumes, particularly when involving increasingly complex and opioid-tolerant chronic pain patients, pose significant challenges for perioperative management.

Inpatient hospital pain services^3^^,^^4^ can be of tangible benefit for these complex pain patients.^5^  Miaskowski  and colleagues found that patients cared for by an acute pain service were discharged sooner, had lower pain intensity scores, lower incidences of pruritis, sedation and nausea compared to those without the anesthesia service involvement.^5^  With a growing number of surgical cases, pain management consultations have increased to a number that is not always sustainable, leading some hospitals to adopt a nurse care model.^6^

There is minimal medical literature assessing the types of pain management consultations.^4^ A local and unpublished study on resource utilization at Mayo Clinic Rochester demonstrated that orthopedic surgeons place roughly 40% of all surgical specialty consultation requests for acute postoperative pain management.  The majority of these consultations are requests for pharmacologic analgesia management, such as titrating oral narcotic medications.  A smaller number of these requests are focused on interventional pain procedures, such as joint injections. Since not all hospitals have dedicated inpatient pain services, this places additional pressure on surgeons in the perioperative period, making pain education for these specialties even more important.

To improve postoperative pain management, the inpatient pain medicine service at Mayo Clinic, a subspecialized physician practice with credentials specifically in Pain Medicine, founded an educational seminar for the incoming orthopedic surgery residents.  The purpose of this program was to optimize their understanding of the role that the inpatient pain service plays in managing postoperative pain, as well as to educate them in the founding principles of pharmacologically-directed pain management.

### Educational Seminar

In 2011, the Orthopedic Staff Leadership Committee (OSLC) at the Mayo Clinic, which consists of a core group of staff nurses and nursing leadership, participated in an educational seminar with new orthopedic residents to help guide them through the dynamic perioperative hospital course to ensure safe, satisfactory patient care.  These residents received clinical education from various hospital groups, including physical and occupational therapy, social services and pharmacy.  The seminars guided them through clinical techniques, such as surgical wound management, pressure ulcer prevention, and proper application of splints and casts.  As an extension of this training, the OSLC, in conjunction with the Anesthesiology Division of Pain Medicine, developed a seminar to orient the resident trainees to common strategies of postoperative pain management.

The training seminar consists primarily of a one-day, three-hour discussion and didactic lecture. This lecture is presented by a core faculty member of the division of Pain Medicine. The core principles it addresses are the nuances of the functions of the Anesthesia Acute Pain Management Hospital Service, as well as the pharmacodynamic and pharmacokinetic principles of a number of common medications used in the postoperative setting.  These include medications such as oral and IV narcotics, NSAIDs, Tylenol, topical local anesthetics, and Tramadol.  Specific information on opioid titration, parenteral to oral opioid conversion, and postoperative opioid weaning strategies (once healed from surgery) was also provided.

The content consisted of defining the concept of multimodal analgesia, reviewing opioid pharmacokinetics and pharmacodynamics, demonstrating how to calculate oral morphine equivalents, understanding the utility of regional anesthesia, and reviewing methadone and pharmacokinetics and pharmacodynamics. A pre and post-assessment, audience response system test was given to improve attention and retention.

## Conclusions

Novel surgical residency education programs have been developed to help bridge knowledge gaps in pain management between surgery and anesthesia colleagues. These include web-based interactive curricula, simulation center training, and exposure to cadaveric anatomy labs for procedural skills.^8^^,^^9^^,^^10^ Despite this, there is a paucity of health care literature regarding the utility of educating resident trainees about the role of inpatient pain services or in the basic principles of postoperative pain management.

Formal pain management education in surgical departments could improve acute pain management of post-surgical patients. Better pain management improves the overall quality of care, patient satisfaction, and provider satisfaction.  As a secondary outcome, the pain service could focus their skills and attention on patients with more complex pain management issues and eliminate costly consults ordered for more basic considerations applicable to routine patient scenarios.  For example, if patients routinely had their medications optimized prior to arrival to the hospital floor, one could reasonably expect better pain control and more efficient use of perioperative resources to address other medical concerns.  Since dedicated inpatient pain services are not universally available, while large numbers of chronic pain patients continue to grow in number, there is an increasing need for physicians of all specialties to acquire fundamental pain medicine knowledge. While resident education is not standardized between programs in the United States, similar teaching models could very well be beneficial and easily implemented in other surgical training programs.

### Conflict of Interest

The authors declare that they have no conflict of interest.
